# Claudin 4 in pancreatic β cells is involved in regulating the functional state of adult islets

**DOI:** 10.1002/2211-5463.12735

**Published:** 2019-11-23

**Authors:** Hongtu Li, Abraham Neelankal John, Takahiro Nagatake, Yoko Hamazaki, Fang‐Xu Jiang

**Affiliations:** ^1^ Islet Cell Development Program Faculty of Medical Science Harry Perkins Institute of Medical Research University of Western Australia Nedlands WA Australia; ^2^ Department of Immunology and Cell Biology Graduate School of Medicine Kyoto University Japan; ^3^ Present address: Centre for reproductive medicine Ciming Boao International Hospital Qionghai Hainan 571434 China; ^4^ Present address: Key Laboratory of Genetics and Reproduction Health of National Health and Family Planning Commission Liaoning Province Research Institute of Family Planning Shenyang 110031 China

**Keywords:** β cells, bioinformatics analysis, claudin 4, dedifferentiation, functional state

## Abstract

The functional state (FS) of adult pancreatic islets is regulated by a large array of regulatory molecules including numerous transcription factors. Whether any islet structural molecules play such a role has not been well understood. Here, multiple technologies including bioinformatics analyses were used to explore such molecules. The tight junction family molecule *claudin 4* (*Cldn4*) was the highest enriched amongst over 140 structural genes analysed. *Cldn4* expression was ~75‐fold higher in adult islets than in exocrine tissues and was mostly up‐regulated during functional maturation of developing islet cells. *Cldn4* was progressively down‐regulated in functionally compromised, dedifferentiating insulin‐secreting β cells and in db/db type 2 diabetic islets. Furthermore, the genetic deletion of *Cldn4* impaired significantly the FS without apparently affecting pancreas morphology, islet architectural structure and cellular distribution, and secretion of enteroendocrine hormones. Thus, we suggest a previously unidentified role for Cldn4 in regulating the FS of islets, with implications in translational research for better diabetes therapies.

## Graphical abstract

Claudin 4 (Cldn4) is developmentally up‐regulated in developing pancreatic islet cells, is involved in sustaining the functional state (FS) in mature islets and is pathologically down‐regulated to compromise the FS in diabetic β cells.

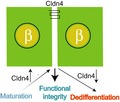

**Abbreviations**
*Cldn4*: *claudin 4*
ESC: embryonic stem cell
FS: functional state
GIP: insulinotropic polypeptide
GLP1: glucagon‐like peptide‐1
TJ: tight junction

A large array of critical regulatory molecules such as transcription factors 1 have been well documented to regulate functional state (FS) of pancreatic endocrine islets of Langerhans that sustain normal glucose homeostasis of the body, mainly via hormones from insulin‐secreting β cells and glucagon‐secreting α cells. To regulate this FS, the islets have evolved to have many unique features including being structurally spread throughout the exocrine pancreas. They do not have their own basement membrane 2 but are closely in contact with that of intra‐islet capillaries 3, 4. In viewing the unique structural arrangement of the pancreatic endocrine tissue, we hypothesize that undiscovered structural molecules are important in regulating islet FS. This study aims to study such molecules.

We have previously demonstrated that functional genes can be identified with the effective bioinformatics analyses of transcriptomic data sets 5, 6. Using the transcriptome data sets and other approaches, we demonstrated the involvement of previously understudied claudin (*Cldn*) family genes of tight junctions (TJs) in the islets. TJs between neighbouring epithelial cells have important biochemical and physiological roles in multiple organs by selective and critical permeability to important compounds, in addition to working as a structural barrier by forming strands against unrestricted paracellular passaging 7. Surprisingly, the expression and function of TJ molecules in the islets of Langerhans are largely unknown.

Cldn family molecules are the tetraspan transmembrane proteins of TJs, forming a structural barrier between the apical and basal portions of epithelial cellular sheets 8. This family consists of at least 28 members in mice and humans. Cldns are classically expressed in epithelium and are categorized as cation‐selective, anion‐selective and water‐permeable channels, and charge‐selective barriers. The distribution of Cldns varies from one tissue type to another 9: for example, Cldn1 regulates permeability in the epidermis 10, Cldn5 does so in the blood–brain barrier 11, Cldn11 in the myelin and Sertoli cells 12, Cldn14 in inner ears 13 and Cldn18 in the stomach 14.

Cldn4 is a member of charge‐selective Cldns, usually partnering with Cldn8 and Cldn12 9. Cldn4 is prominently expressed in the lung, intestinal and kidney‐collecting tubular epithelia 8, the urinary bladder and skin 15 and the atypical and nonpolarized epithelial cells such as thymic epithelial cells 16, 17. Though highly expressed, Cldn4 seems not to play a major role on the physiology of the lung 18. Cldn4 is functionally involved in the generation of thymus CD4/CD8 single positive T lymphocytes 19. Gene knockout experiments demonstrate that *Cldn4* is critical for renal chloride (Cl^−^) reabsorption and blood pressure regulation 20, 21. Cldn4 was previously detected by immunofluorescence in the rat pancreatic tissue as well as in the islets of Langerhans 22. However, no major pathophysiological effect on energy metabolism has been documented on any *Cldn* gene. Here, we show with a number of approaches that Cldn4 in the mouse pancreatic islets is associated with regulating FS of the islets, implicating in translational research for better diabetes therapies.

## Materials and methods

### Mouse lines

The *Cldn4*‐deleted, floxed *Cldn4* and CAG‐Cre mice 23 were bred onto a C57BL/6 background for at least 10 generations. PCR‐based genotyping for Cldn4^+/−^ and Cldn4^−/−^ mouse lines was described elsewhere 23. Cldn4^−/−^, Cldn4^+/−^, floxed *Cldn4* and CAG‐Cre (the latter two lines along with the C57BL/6 designated as Cldn4^+/+^) mouse lines and the type 2 diabetes model db/db mice provided by Jackson Laboratory (Mount Desert Island, ME, USA) were maintained in a 22 ± 1 °C, 12:12 light/dark cycle environment with free access to food and water and used at 8–12 weeks of age.

### Compliance with Ethical Standards

All applicable international, national and/or institutional guidelines for the care and use of animals were followed, namely the Animal Ethics Committees of the University of Western Australia, Australia and Kyoto University, Japan, approved the use of experimental animals.

### MIN6 cells

Culture, maintenance and passage of MIN6 cells were described previously 24.

### Isolation of adult islets

Islets of Langerhans were isolated from euthanized (cervical dislocation) 8‐ to 12‐week‐old C57BL/6 mice, 12‐week db/^+^ mice and db/db diabetic mice as described recently 5. Briefly, the pancreas was injected via the bile duct with collagenase P solution (Sigma, Melbourne, Vic., Australia, 1.2 mg·mL^−1^ dissolved in Hanks’ balanced salt solution containing 2 mm Ca^2+^ and 20 mm HEPES). Islets and exocrine layers were isolated by density gradient Histopaque (Sigma) centrifugation, washed and hand‐picked islets for RNA.

### Glucose‐stimulated insulin secretion assay

Glucose‐stimulated insulin secretion assay was performed essentially as described 25. Briefly, the indicated passaged MIN6 cells were washed twice with warm PBS. After pre‐incubation with the Krebs–Ringer buffer at 37 °C for 90 min, the cells were incubated at 37 °C for 60 min with basal D‐glucose (2.75 mm) or stimulus D‐glucose (27.5 mm). Then, each conditioned medium was collected to determine the insulin concentration using a mouse insulin ELISA kit (Mercodia AB, Uppsala, Sweden). Subsequently, the culture was trypsinized and the number of MIN6 cells was determined with a haemocytometer.

### Oral glucose tolerance test and serum incretin concentrations

After overnight fasting, mice were orally administered 10% glucose (2 g·kg^−1^ body weight) and blood glucose levels were measured with tail vein blood using OneTouch UltraVue (Johnson & Johnson K.K., Nishikanda Chiyoda‐Ku, Japan). Serum glucose‐dependent insulinotropic polypeptide (GIP) and glucagon‐like peptide‐1 (GLP1) concentrations were determined with Bio‐Plex (Bio‐Rad, Shinagawa‐ku, Tokyo, Japan), according to the manufacturer's instruction.

### Generation of endodermal cells

Endodermal cells were generated from directed differentiation of undifferentiated mouse embryonic stem cell (ESC) W9.5 line as we described previously 6.

### Bioinformatics analyses

Bioinformatics analyses of transcriptome data sets were performed on published data sets generated from ESCs and isolated adult mouse islets and during differentiation of islet progenitors 25. Gene mining was performed as described previously 6, 26. Briefly, the differential expression of genes (*P* ≤ 0.05; −1 ≤ log_2 _≥ 1) based on bioinformatics contrast between ESC and islet data sets and other contrasts were analysed using the Limma package in the ‘r’ environment (http://bioinf.wehi.edu.au/limma). We will provide the data sets on request.

### Indirect immunofluorescence

Rat anti‐claudin 4 antibodies were purchased from Millipore (Castle Hill, NSW, Australia) and Abcam (Cambridge, UK), respectively. Biotinylated anti‐human insulin and rabbit anti‐glucagon antibodies were purchased from R&D Systems (Minneapolis, MN, USA). The antibody sources and staining procedures for laminin and nidogen 1 were described previously 2. Cells and pancreases from all genotypes were fixed in 4% paraformaldehyde and the latter processed for histological sections. After dewaxing and rehydration, tissue sections along with cell preparations were stained with primary antibodies and reacted with streptavidin FITC (BD, Bergen County, NJ, USA) and Texas Red anti‐human, anti‐rat or anti‐mouse (Vector Labs, Burlingame, CA, USA) as we described previously 27. Slides were observed and microphotographed with the inverse IX71 Olympus fluorescence microscope (Olympus, Tokyo, Japan).

### Gene expression analyses by qRT‐PCR

Total RNA was extracted from the epididymal fat, liver, kidney and skeletal muscles in euthanized (cervical dislocation) adult C57BL/6 mice and other indicated cells with RNeasy Plus Mini Kit (Qiagen Science, Melbourne, Vic., Australia) or the TRIzol‐based method and quantified by a NanoDrop ND‐1000 Spectrophotometer (Australian Biolab group, Melbourne, Vic., Australia) as described previously 26. RNA (200–400 ng) was reverse‐transcribed with reverse transcriptase to cDNA in 40 μL, 1 μL of which (5–10 ng RNA/reaction, without reverse transcriptase as a negative control) was amplified by qRT‐PCR analysis essentially as we described 26. Primer sequences are presented in Table 1.

**Table 1:** Sequences for qRT‐PCR primers.

Gene	Forward primer 5′ → 3′	Reverse primer 5′ → 3′	Annealing temperature (°C)	Product (bp)
*Acta1*	caatcgtgctgtggttgcag	ggagcaaaacagaatggctgg	60	191
*Cldn3*	ggagtgcttttcctgttggc	cgtagtccttgcggtcgtag	60	295
*Cldn4*	ccaagtcatggtgtgctgag	cactgggctgcttctaggtc	60	217
*Fev*	cggcgtctactcttccctgt	catctccgacgggatctggc	60	191
*FoxO1*	agccgcgcaagaccag	ttgaattcttccagcccgcc	60	195
*Hnf4a*	ggatatggccgactacagcg	agatggggacgtgtcattgc	60	100
*IA1*	gccacccgtctgagaataga	ggagtcacagcgagaagacc	60	231
*Lama1*	cggcgcgtaaagatttccag	ctcctgggtcttgcttccag	60	290
*Lamb1*	gccgtcctaatgtggttgga	agctgggaaagccccaatac	60	210
*Isl1*	cccgggggccactatttg	cgggcacgcatcacgaa	60	397
*MafA*	atcatcactctgcccaccat	agtcggatgacctcctcctt	60	208
*Mmp2*	ggctctgtcctcctctgtag	tgccctcctaagccagtct	60	296
*Mycl1*	gagaacggctggagagagtg	ttcaccttcagaatcgctggg	60	200
*NeuroD*	cttggccaagaactacatctgg	ggagtagggatgcaccgggaa	60	228
*Nid1*	atcagcaccatccctgaaac	tcaataccgctgaactgctg	60	206
*Pax4*	ccacctctctgcctgaagac	cccacagcatagctgacaga	60	236
*Pou3f4*	gctgcctcgaatccctacag	cagttgcagatcttcgcgtc	60	261
*Rfx6*	gcttgctggtctaccctgag	catcatctgcgtgatgctct	60	233
*Rps18*	tgtggtgttgaggaaagcag	tcccatccttcacatccttc	60	155
*Snail1*	agttgactaccgaccttgcg	tgcagctcgctatagttggg	60	128
*Snail2*	ggaccgttatccgcccg	tatggggaaataataactgtgtgtg	60	130

The power SYBR Green PCR Master Mix‐based protocol was utilized, and all quantifications were normalized to the internal 18s rRNA level, as described recently 5. Briefly, cDNA was amplified with PCR: 95 °C for 10 min, followed by 40 cycles of 95 °C for 15 s and 60 °C for 1 min. The number of cycles of threshold (*C*_t_) was measured with an ABI Prism 7900HT Sequence Detection System (Applied Biosystems, Foster City, CA, USA) or a Rotor‐Gene RG‐3000 (Corbett Research, Sydney, NSW, Australia).

### Statistical analysis

Experiments were performed in at least three biological repeats. Data are expressed as mean ± standard deviation (SD). Statistical differences between groups are analysed with nonparametric, unpaired Mann–Whitney *U* tests or Student's *t* tests in samples with numerous biological repeats.

## Results

### Bioinformatics analyses identified unique pancreatic islet genes

**Figure 1:**
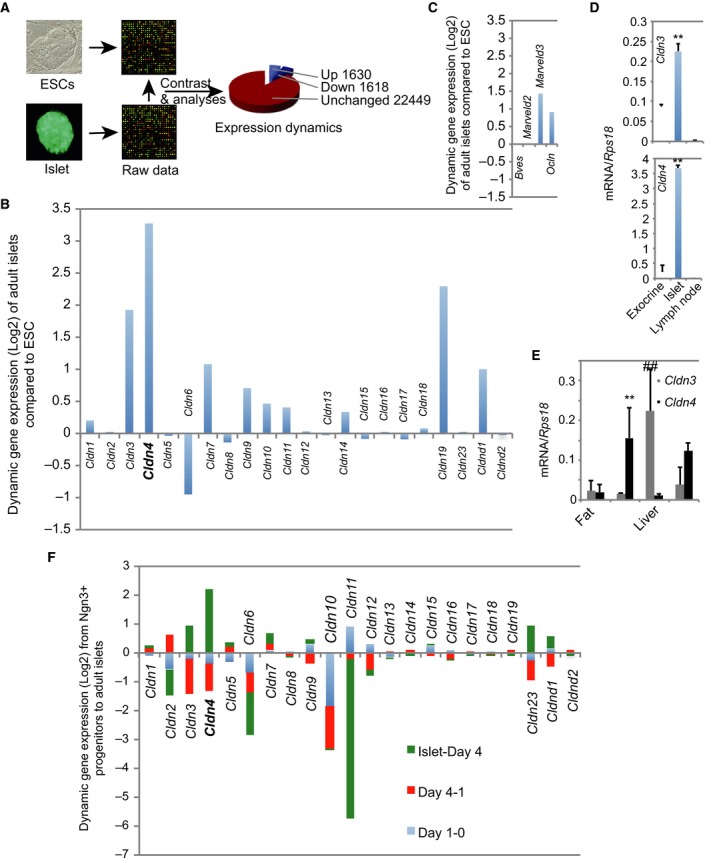
*Claudin 4* is highly expressed in adult functional islet cells. (A) Scheme depicted of the process of analysing transcriptomic data sets. Analyses of transcriptomic data sets generated from RNA extracted from ESCs and isolated adult islets. (B) Bioinformatics contrast analyses of the TJ claudin (Cldn) family genes with *Cldn4* bolded and enlarged. (C) Differential contrast analyses of the TJ‐associated genes of occludin family. (D) qRT‐PCR analysis of adult islet *Cldn3* and *Cldn4*. RNA was extracted from the pancreatic exocrine tissue (a epithelial tissue), the isolated adult islets and lymph nodes (a mesenchymal tissue). Data presented as mean ± SD, *n* = 3, **P < 0.01 compared to exocrine or lymph node (Mann–Whitney *U* tests). (E) qRT‐PCR analysis of *Cldn3* and *Cldn4* in selected metabolic tissues. RNA was extracted from fat, renal tissue, the liver and skeletal muscle. Data presented as mean ± SD, *n* = 3, **P < 0.01 compared to fat or the liver; ^##^ *P* < 0.01 compared to all other tissues (Mann–Whitney *U* tests). (F) *Claudin 4* (bolded and enlarged) is predominantly up‐regulated during functional maturation of islet β cells. Bioinformatics analyses of the claudin (Cldn) family genes in global gene expression data sets 25 during *in vitro* differentiation of islet progenitors for 0 (Day 0, namely undifferentiated islet progenitors)‐, 1 (Day 1, namely 1‐day differentiation of islet progenitors)‐ and 4 (Day 4, namely 4‐day differentiation of islet progenitors)‐day differentiation and using adult islets (islet) as the reference.

To identify structural molecules that may regulate islet FS, we first conducted bioinformatics analyses to survey unique genes in adult pancreatic islets on the published global transcriptional data sets 6, 25. A bioinformatics contrast of the data sets generated from isolated functional islets to undifferentiated pluripotent ESCs (as a baseline) showed that there were 1618 and 1630 genes negatively and positively enriched (Log_2_ scale), respectively (Fig. 1A). Here, we only focused our analyses on genes that encode structural molecules for TJs, the basement membrane and mesenchymal tissue. Genes for the latter were analysed as the adult pancreatic islets are reported to have mesenchymal features 28.

### Tight junction *claudin 4* gene was highly enriched in adult functional islets

In the bioinformatics contrast, there were 22 single exon TJ *Cldn* genes. Whereas majority of *Cldn* genes (*Cldn1*,* Cldn5*,* Cldns7‐9* and *Cldns12‐18*) were undetectable, *Cldn6* was strikingly negatively enriched (Fig. 1B). Interestingly, we revealed that several TJ genes *Cldn3* and *Cldn19* and in particular *Cldn4* were significantly enriched (Fig. 1B). *MarvelD3*, the TJ‐associated member of occludin family, was also enriched (Fig. 1C), but not essential for the formation of the TJs 29.

To confirm the high enrichment of *Cldn4* in functional islets, qRT‐PCR analyses were conducted with RNA extracted from adult islets compared to that from pancreatic exocrine cells, a typical epithelial tissue and from the lymph nodes, a mesenchymal tissue. *Cldn4* in adult islets was indeed highly enriched by approximately 75‐fold, whereas *Cldn3* only twofold to threefold (Fig. 1D), compared to the exocrine cells. We also surveyed their expression in peripheral glucose metabolic tissues such as fat, liver and skeletal muscle using the renal tissue as the positive control 8. *Cldn4* expression was approximately 20‐fold to 30‐fold higher in the islets than in the kidney and the skeletal muscle (Fig. 1D,E); *Cldn3* was, however, mainly expressed in the liver, whereas both genes were not detected in fat (Fig. 1E).

### *Claudin 4* was highly expressed in adult functional islets

To determine at what developmental stage *Cldn4* is highly enriched, we performed bioinformatics contrasts of published transcriptome data sets generated from *in vitro* differentiation of purified mouse islet progenitors into immature insulin‐secreting cells and from isolated adult islets 25. Indeed, we demonstrated that *Cldn4* and to a less extent *Cldn3* and *Cldn23* were significantly enriched during functional maturation of islet cells (Fig. 1F). Taken together, we conclude that Cldn4 is the highly expressed TJ molecule amongst the Cldn family in adult islets.

### Claudin 4 was down‐regulated in functionally compromised dedifferentiated β cells

**Figure 2:**
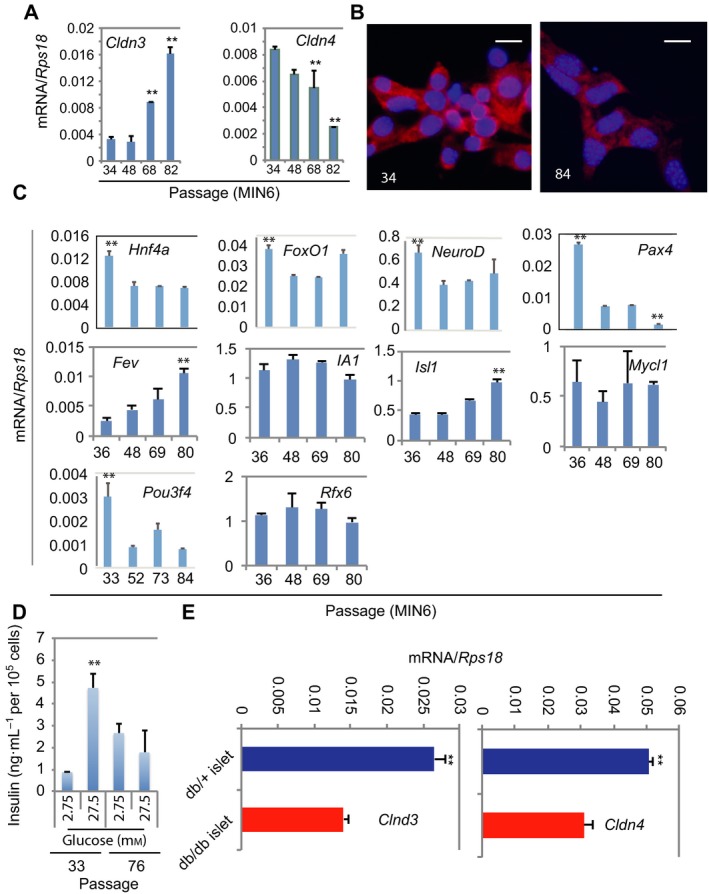
*Claudin 4* is down‐regulated during dedifferentiation of functional βcells. (A) qRT‐PCR analysis of *Cldn3* and *Cldn4*. RNA was extracted from MIN6 cells at passages 34, 48, 68 and 82. (A, B) Data presented as mean ± SD, *n* = 3, **P < 0.01 compared to all other passages (Mann–Whitney *U* tests). (B) Immunofluorescence analysis. MIN6 cells at passages 34 and 84 were stained with anti‐Cldn4 (red) and the DNA dye DAPI (blue). Scale bar = 20 μm. (C) Glucose‐stimulated insulin secretion analysis. Indicated passaged MIN6 cells were exposed to basal glucose (2.75 mm) or stimulus glucose (27.5 mm) for the determination of insulin concentrations. Data presented as mean ± SD, *n* = 3, **P < 0.01 compared to the basal glucose condition (Mann–Whitney *U* tests). (D) qRT‐PCR analysis of selected transcription factor genes associated with islet function and differentiation. RNA was extracted from MIN6 cells at passages 36, 48, 69 and 80. Data presented as mean ± SD, *n* = 3, **P < 0.01 compared to all other passages (except *FoxO1* and *NeuroD* not vs passage 80) (Mann–Whitney *U* tests). (E) qRT‐PCR analysis of *Cldn3* (grey bars) and *Cldn4* (black bars). RNA was extracted from isolated db/+ and db/db diabetic islets. Data presented as mean ± SD, *n* = 3, **P < 0.01 compared to db/db diabetic islets (Mann–Whitney *U* tests).

If the Cldn4 molecule is a critical regulator, we hypothesized that reduction of its expression or genetic deletion will compromise the FS of adult pancreatic islets. We first investigated the dynamic changes of Cldn4 expression in a characterized cellular model of dedifferentiation, the passaged MIN6 β cells 24. β‐cell dedifferentiation is broadly defined as becoming insulin‐negative cells, losing function of GSIS, re‐expressing markers of islet progenitors and even transdifferentiating to other islet cell types 24, 30. The expression of Cldn4 molecule was progressively and significantly (*P* < 0.001) down‐regulated at mRNA (Fig. 2A) but not clearly reduced at protein (Fig. 2B) levels in dedifferentiating MIN6 cells 24, whereas *Cldn3* was significantly up‐regulated in dedifferentiating MIN6 cells (*P* < 0.001). The significance of different expression profiles between *Cldn3* and *Cldn4* deserves further investigation. To shed mechanistic lights, we investigated dynamic changes of many progenitor and functional transcription factor genes. The functional transcription factor genes *Hnf4a*,* FoxO1*,* NeuroD*,* Pax4* and *Pou3f4* (also known as *Brn4*) expression was significantly (*P* < 0.001) down‐regulated at early dedifferentiating stage. The expression of islet progenitor transcription factor genes *Fev* and *Isl1* was progressively (*P* < 0.001) up‐regulated, whereas *IA1* (also known as *Insm1*), *Mycl1* and *Rfx6* was unchanged (Fig. 2C). We also confirmed that dedifferentiated MIN6 cells significantly lost their glucose‐stimulated insulin secretion (Fig. 2D), consistent with previous report 24.

To provide evidence whether the down‐regulation of *Cldn4* takes place in the dedifferentiated primary β cells, islets were isolated and analysed from the well‐characterized type 2 diabetes model db/db mice that have dedifferentiated β cells 24, 31. We showed that *Cldn4* was down‐regulated approximately 60% in dedifferentiated db/db islets (Fig. 2E). Collectively, these data suggested that Cldn4 involves in regulating FS in mature β cells and reduction of which could mark the dedifferentiation of β cells.

### *Claudin 4* deletion compromised glucose tolerance

**Figure 3:**
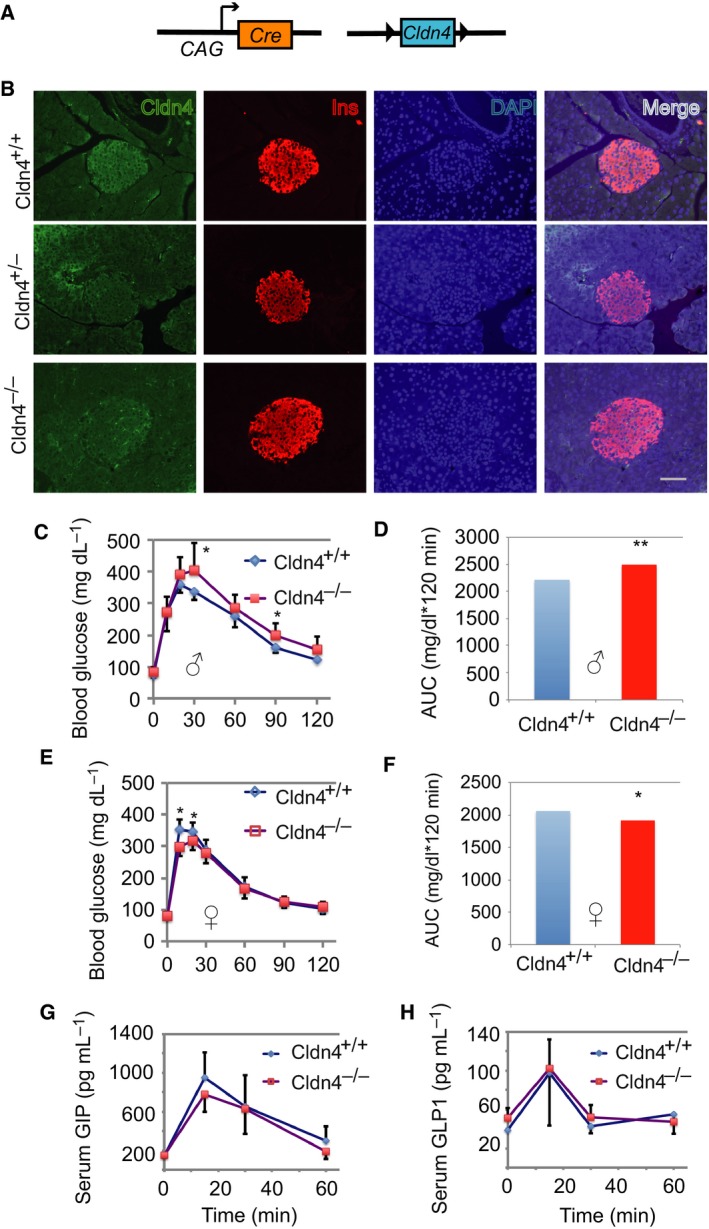
*Cldn4* deletion compromises glucose tolerance. (A) Schema showing the *Cldn4* deletion strategy. The chicken β‐actin promoter/enhancer coupled with the cytomegalovirus immediate‐early enhancer (CAG) driving Cre‐mediated deletion of the floxed *Cldn4*. (B) Immunofluorescence analysis. Pancreas sections from Cldn4^+/+^, Cldn4^+/−^ and Cldn4^−/−^ mice were stained with anti‐Cldn4 (green) and anti‐insulin (Ins, red), and the DNA dye DAPI (blue). Microphotographs were taken under a microscope. Scale bar = 50 μm. (C) Oral glucose tolerance test (OGTT) in males. Cldn4^+/+^ (*n* = 9) and Cldn4^−/−^ (*n* = 8) adult mice were examined. Blood glucose concentrations were determined in the tail vein using an OneTouch UltraVue glucose metre. (D) AUC analysis of OGTT in (C). (E) OGTT in females. Cldn4^+/+^ (*n* = 12) and Cldn4^−/−^ (*n* = 13) adult mice were examined. Blood glucose concentrations were determined as in (C). (F) AUC analysis of OGTT in (E). (F) Serum glucose‐dependent GIP concentrations. Adult male Cldn4^+/+^ (*n* = 4) and Cldn4^−/−^ (*n* = 4) mice were used. (G) GIP concentrations were determined with Bio‐Plex assays. (H) Serum glucagon‐like polypeptide‐1 (GLP1) concentrations. Adult male Cldn4^+/+^ (*n* = 3) and Cldn4^−/−^ (*n* = 3) mice were tested. GLP1 concentrations were determined with Bio‐Plex assays. (C–H) Data presented as mean ± SD, *P < 0.05 and **P < 0.001 compared to Cldn4^+/+^ (Student's *t* tests).

We hypothesized that if the dynamic changes of Cldn4 expression were the consequence of β‐cell maturation or dedifferentiation, *Cldn4* deletion will not compromise the islet FS. To test this hypothesis, we analysed the well‐established genetic mouse model in which *Cldn4* was globally removed 23, diagrammatically depicted in Fig. 3A. The global knockout model was used because the effect of *Cldn4* in liver, fat and skeletal muscles was negligible as its expression was undetectable in liver and fat and approximately 30‐fold lower than in the islets (Fig. 1D,E). Both Cldn4^+/−^ and Cldn4^−/−^ mice were born in a normal Mendelian ratio and developed physically indistinguishable from Cldn4^+/+^ mice. The deletion of *Cldn4* was previously confirmed by Southern plot analysis in Cldn4^−/−^ mouse ESCs 23. We here also showed that the expression of Cldn4 protein was undetectable or dramatically reduced in *Cldn4‐*deleted β cells by immunofluorescence analysis (Fig. 3B), indicating that *Cldn4* was effectively inactivated in the mutant islets.

We then performed oral glucose tolerance test, as the enteroendocrine cells express a high level of Cldn4 32 and secrete incretins that are also modulators of glucose homeostasis in Cldn4^+/+^ mice 33. After overnight fasting, Cldn4^−/−^ mice were administered oral glucose at 2.0 g·kg^−1^ and glucose concentrations measured. The Cldn4^−/−^ mice showed a sex difference in responding to glucose challenge. Cldn4^−/−^ males had modest but significantly (*P* < 0.05) higher blood glucose concentrations at 30 and 90 min (Fig. 3C). This was confirmed by a highly significantly (*P* < 0.01) increase of the area under the curve (AUC) in Cldn4^−/−^ males compared to Cldn4^+/+^ males (Fig. 3D). Surprisingly Cldn4^−/−^ females were modest but significantly (*P* < 0.05) more sensitive to glucose metabolism 10 and 20 min after the glucose challenge (Fig. 3E). This was confirmed by a significantly (*P* < 0.05) decrease of the area under the curve (AUC) in Cldn4^−/−^ females compared to Cldn4^+/+^ females (Fig. 3F). Hereafter, we only described phenotypes from male Cldn4^−/−^ mice.

We hypothesized, if abnormal blood glucose concentrations in these mice are caused by abnormal function of enteroendocrine cells, that the blood concentrations of key gut hormones will be abnormal due to the deficiency of *Cldn4* in these cells 32. Nevertheless, the concentrations of glucose‐dependent insulinotropic polypeptide (also known as gastric inhibitory polypeptide, GIP, Fig. 3G) and glucagon‐like polypeptide‐1 (GLP1, Fig. 3H) were similar between Cldn4^+/+^ and Cldn4^−/−^ mice, suggesting that the deficiency of *Cldn4* does not affect the secretion of these enteroendocrine hormones. Taken together, these data show that Cldn4 deletion in adult male islets is associated with the glucose intolerance and compromised islet FS observed.

### *Claudin 4* deletion did not affect islet architecture

**Figure 4:**
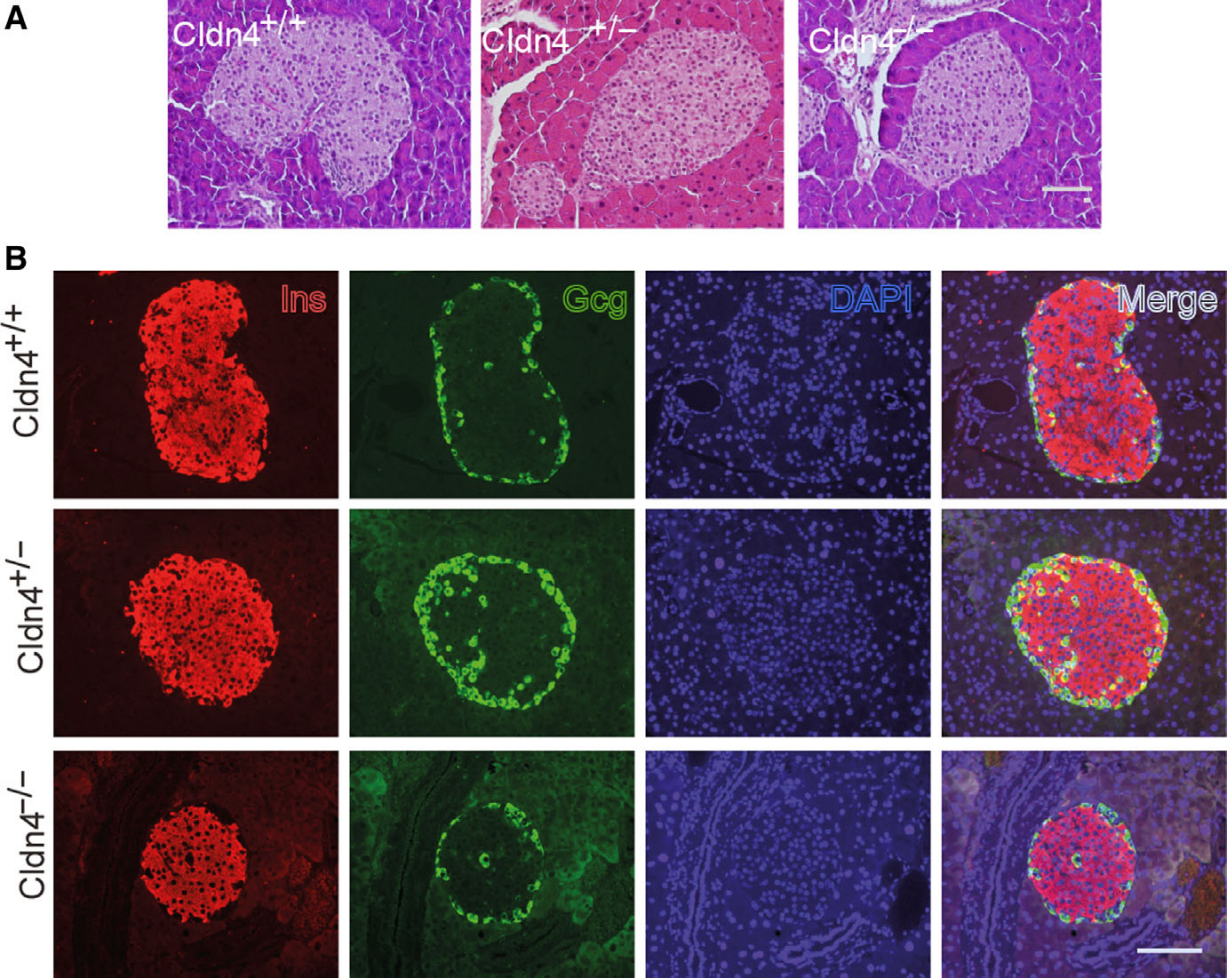
*Claudin 4* deletion appears not affecting islet architecture. (A) Histological analyses. Pancreas sections from adult Cldn4^+/+^, Cldn4^+/−^ and Cldn4^−/−^ mice were stained with haematoxylin and eosin (H&E). Scale bar = 50 μm. (B) Immunofluorescence analysis. Pancreas sections from Cldn4^+/+^, Cldn4^+/−^ and Cldn4^−/−^ mice were stained with anti‐insulin (Ins, red) and anti‐glucagon (Gcg, green) and the DNA dye DAPI (blue). Representative microphotographs were taken under a microscope. Scale bar = 50 μm.

Cldn4 is a key member of the TJ family molecules, deletion of which in male pancreas may disrupt the islet architecture and lead to an inadequate cellular distribution, that could impair the FS of adult islets 34, 35. We thus performed morphological and immunofluorescence analyses in adult male *Cldn4*‐deleted pancreases. Interestingly, pancreas morphology and the islet architecture were similar and apparently unchanged in Cldn4^+/−^ and Cldn4^−/−^ compared to Cldn4^+/+^ mice (Fig. 4A). Furthermore, the cellular distribution was also apparently similar amongst Cldn4^+/+^, Cldn4^+/−^ and Cldn4^−/−^ islets with β cells at the core and α cells at the mantle (Fig. 4B). Combining with the observations that Cldn4 is up‐regulated during maturation and down‐regulated during dedifferentiation of β cells, we suggest that *Cldn4* deletion compromised islet FS. Finally, we examined whether basement membrane and mesenchymal genes are also involved in sustaining islet FS.

### Basement membrane genes were absent in adult islets

**Figure 5:**
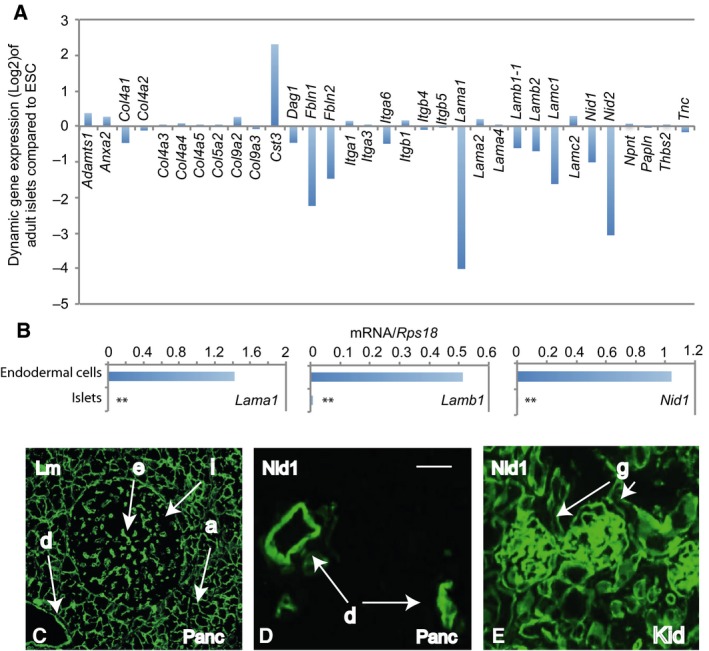
Basement membrane genes are undetectable in adult islets. (A) Differential contrast expression of selective basement membrane genes in the transcriptomic data sets between the ESCs and adult islets. (B) qRT‐PCR analysis for *Lama1*,* Lamb1* and *Nid1*. RNA was extracted from isolated adult islets and ESC‐derived endodermal cells, the latter were used as a positive control. Data presented as mean ± SD, *n* = 3, **P < 0.01 compared to endodermal cells (Mann–Whitney *U* tests). (C–E) Immunofluorescence analyses from the adult mouse pancreas and kidney were stained for laminin (Lm, C) and nidogen 1 (Nid1, D–E). Scale bar = 50 μm. a: the acinar cell basement membrane; d: the ductal epithelial basement membrane, e: the endothelial basement membrane, g: the kidney glomerular basement membrane and i: islet.

Encoding important structural proteins surrounding the islet cells, basement membrane genes were also analysed. Bioinformatics analyses showed that several laminin chain genes *Lama1*,* Lamb1* and *Lamc1*, and key linkage genes *Fbln1*,* Fbln2*,* Nid1* and *Nid2* were all negatively enriched in mouse islet cells compared to that of ESCs (Fig. 5A). Supportively, qRT‐PCR did not detect meaningful expression of *Lama1*,* Lamb1* and *Nid1* in isolated mouse islets (Fig. 5B). Immunofluorescence analyses verified that laminin and laminin linking molecule nidogen 1 proteins were undetectable around islet cells, though present in a variety of endo‐ and epithelial basement membranes (Fig. 5C–E). Taken together, no basement membrane molecule was likely to play a role in sustaining islet FS.

### Fewer mesenchymal genes expressed in adult islets

**Figure 6:**
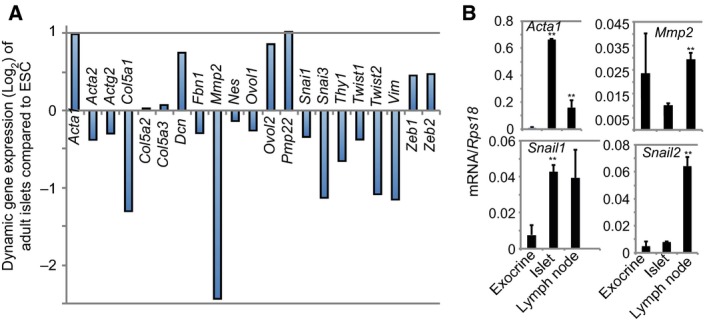
Fewer mesenchymal genes are detectable in adult islets. (A) Differential contrast expression of selective mesenchymal genes in the transcriptomic data sets between the ESCs and adult islets. (B) qRT‐PCR analysis for *Acta1*,* Mmp2*,* Snail1* and *Snail2*. RNA was extracted from the pancreatic exocrine (an epithelial tissue), the isolated adult islets and lymph nodes (a mesenchymal tissue). Data presented as mean ± SD, *n* = 3, **P < 0.01 compared to exocrine or islets (Mann–Whitney *U* tests).

Amongst a selective cohort of 21 mesenchymal genes 36, the expression of the mesenchymal transcription factor genes *Snail3* and *Twist2* and several others *Col5a1*,* Pmp2* and *Vim* was negatively enriched though *Acta1*,* Dcn*,* Ovol2* and *Pmp22* were positively enriched over twofold in adult functional islets compared to that of ESCs (Fig. 6A). qRT‐PCR analysis confirmed that the expression of *Acta1* and *Snail1* was significantly higher in islets than in the exocrine tissue (Fig. 6B). Collectively, the modest enrichment of several mesenchymal genes in the adult islets did not clearly support the notion that these genes play a role in regulating islet FS.

## Discussion

**Figure 7:**
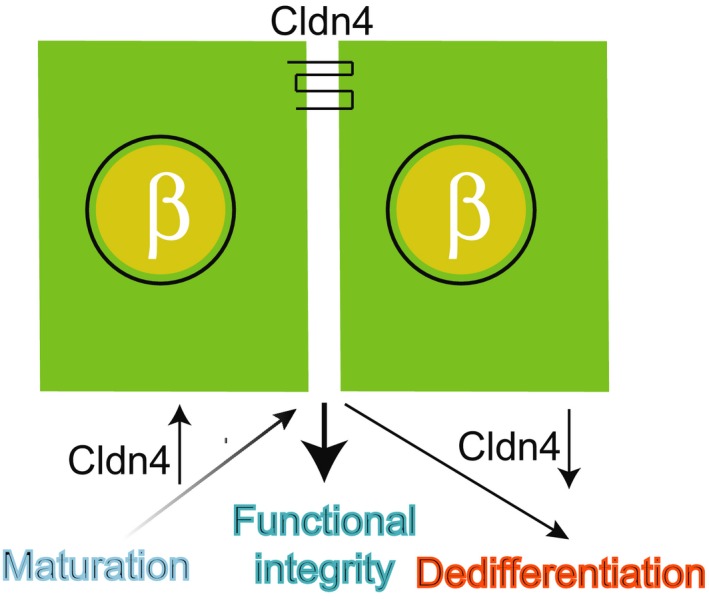
Summary of Cldn4 function. Claudin 4 (Cldn4) is developmentally up‐regulated in developing pancreatic islet cells, is involved in sustaining the FS in mature islets and is pathologically down‐regulated to compromise the FS in diabetic β cells.

We provided multiple pieces of evidence for the first time that the highly expressed TJ molecule Cldn4 may be involved in regulating the FS of the pancreatic insulin‐secreting β cells with implications in translational research for better diabetes therapies. First, we showed that *Cldn4* is mostly up‐regulated during which differentiated β cells are functionally maturated. Second, the expression of *Cldn4* is down‐regulated when β cells are functionally compromised and undergo dedifferentiation. Third, the expression of Cldn4 is also down‐regulated when type 2 diabetic db/db islets have a overtly impaired FS. Fourth, a modest but significantly impaired FS is detected when *Cldn4* is genetically deleted in mice without clearly disrupting islet architecture and cellular distributions. Finally, the impaired FS in *Cldn4*‐deleted mice was apparently not associated with the incretin metabolism as GIP and GLP1 plasma concentrations were unaffected. The absence of meaningful expression of basement membrane genes in purified adult mouse islets supports our previous report 2. In and around mature pancreatic islets, the observed laminin is located at the endothelial basement membrane, consistent with our previous report 2 and produced from endothelial cells and fibroblasts 37. In summary, the above data collectively suggest that the developmental up‐regulation of Cldn4 involves in islet FS, whereas pathological down‐regulation or genetic deletion of Cldn4 compromises it (Fig. 7).

However, unlike the kidney‐collecting duct 20, islet Cldn4 does not structurally partner with its typical partners, *Cldn8* and *Cldn12* 9, as the latter are undetectable in the functional endocrine pancreas. We speculate that islet Cldn4 also acts as selective and critical physiological ion channels 9. As reported 38, β‐cell Cldn4 may interact with the Cldn4 of adjacent β cells for a coordinated signalling. TJs can receive and convert signals from the cell interior to regulate junction assembly and transduce signals to the cell interior to regulate gene expression and cell response 39. A previous study demonstrated that serum Ca^+2^ concentration in the *Cldn4* null mice was significantly decreased, potentially due to the increased excretion of Ca^+2^ and Cl^−^ in the urine 23. If a similar Ca^+2^ metabolic disorder compromises β‐cell function, glucose intolerance should occur in both sexes of Cldn4^−/−^ mice, but in this case, glucose intolerance was only detected in males. Future definitive experiments including pancreas‐ or β‐cell‐specific deletion of *Cldn4* are required to confirm or refute the observation that Cldn4 involves in regulating the islet FS.

Our study suggests a possibility that the highly up‐regulated TJ Cldn4 molecule works as a maturation biomarker of postnatal insulin‐secreting β cells. Using the biomarker, the fully matured insulin‐secreting cells given rise from pluripotent stem cells would be enriched for a regenerative therapy to high‐risk type 1 diabetic sufferers 40. A monoclonal antibody targeted the extracellular loop of Cldn4 has indeed enabled the enrichment of mouse enteroendocrine cells 32. The β‐cell hormone urocortin 3 41 has been demonstrated to be a β‐cell maturation marker 42 but is difficult to be utilized for the enrichment of matured insulin‐secreting cells.

Data presented point to the possibility that the declining expression of Cldn4 works as a novel biomarker of β‐cell dedifferentiation. β‐cell dedifferentiation has been demonstrated to play a critical role in the development of mouse 31 and human 43 type 2 diabetes, which affects 425 million people worldwide. Identification of such biomarkers would facilitate the investigations of molecular mechanisms of β‐cell dedifferentiation and of therapeutic approaches of the dedifferentiation prevention and of redifferentiation. Dedifferentiated β cells in diabetes have abnormally expressed the mitochondrial enzyme aldehyde dehydrogenase 1 isoform 3A 44 or re‐expressed the fetal islet hormone gastrin 45. β‐cell dedifferentiation can be induced by the genetic deletion of *FoxO1* 31 or *Pax6* 46, 47 transcription factor genes. We here showed that when β cells undergo dedifferentiation, *Cldn4* expression is down‐regulated, associated with activation of several islet progenitor transcription factor genes including *Fev* and *Isl1*. Dedifferentiated β cells also progressively lose protein content but increase mRNA of the nuclear receptor Vdr expression and treatment with Vdr agonists is able to prevent β‐cell dedifferentiation 48. Research is underway to understand how the declined expression of functional transcription factor genes such as *Hnf4a* and *Pax4* and/or the increased expression of progenitor transcription factor genes *Fev* and *Isl1* contribute to the decreasing expression of Cldn4 in dedifferentiated β cells. We noted that *Cldn3* was down‐regulated approximately 50% in db/db islets, but significantly up‐regulated in late dedifferentiating MIN6 cells. Its significance on β‐cell dedifferentiation requires further studies.

In summary, our study suggested that the previously unappreciated TJ molecule Cldn4 is involved in regulating islet FS in adult ß cells and may act as a biomarker of β‐cell maturation. This may be of significance for translational research in establishing stem cell therapy for the diabetes sufferers 49. This report also suggested that a reduction in Cldn4 expression in ß cells is associated with their dedifferentiation. Biomarkers of β‐cell dedifferentiation would impact the translational research for redifferentation therapies of the pandemic type 2 diabetes.

## Conflict of interest

The authors declare no conflict of interest.

## Author contributions

F‐XJ conceived, designed and performed the research and wrote the manuscript; HL, ANJ and TN performed the research and YH partially designed the research and reviewed the manuscript.
